# The IL-12 Cytokine and Receptor Family in Graft-vs.-Host Disease

**DOI:** 10.3389/fimmu.2019.00988

**Published:** 2019-05-08

**Authors:** David Bastian, Yongxia Wu, Brian C. Betts, Xue-Zhong Yu

**Affiliations:** ^1^Department of Microbiology and Immunology, Medical University of South Carolina, Charleston, SC, United States; ^2^Department of Medicine, University of Minnesota, Minneapolis, MN, United States; ^3^Department of Medicine, Medical University of South Carolina, Charleston, SC, United States

**Keywords:** GVHD, signal transduction, GVT, cytokine receptor, cytokine, IL-12 cytokines, IL-12 family cytokine receptors, HCT

## Abstract

Allogeneic hematopoietic cell transplantation (allo-HCT) is performed with curative intent for high- risk blood cancers and bone marrow failure syndromes; yet the development of acute and chronic graft-vs.-host disease (GVHD) remain preeminent causes of death and morbidity. The IL-12 family of cytokines is comprised of IL-12, IL-23, IL-27, IL-35, and IL-39. This family of cytokines is biologically distinct in that they are composed of functional heterodimers, which bind to cognate heterodimeric receptor chains expressed on T cells. Of these, IL-12 and IL-23 share a common β cytokine subunit, p40, as well as a receptor chain: IL-12Rβ1. IL-12 and IL-23 have been documented as proinflammatory mediators of GVHD, responsible for T helper 1 (Th1) differentiation and T helper 17 (Th17) stabilization, respectively. The role of IL-27 is less defined, seemingly immune suppressive via IL-10 secretion by Type 1 regulatory (Tr1) cells yet promoting inflammation through impairing CD4^+^ T regulatory (Treg) development and/or enhancing Th1 differentiation. More recently, IL-35 was described as a potent anti-inflammatory agent produced by regulatory B and T cells. The role of the newest member, IL-39, has been implicated in proinflammatory B cell responses but has not been explored in the context of allo-HCT. This review is directed at discussing the current literature relevant to each IL-12-family cytokine and cognate receptor engagement, as well as the consequential downstream signaling implications, during GVHD pathogenesis. Additionally, we will provide an overview of translational strategies targeting the IL-12 family cytokines, their receptors, and subsequent signal transduction to control GVHD.

## Allogeneic Hematopoietic Stem Cell Transplantation

Allogeneic hematopoietic cell transplantation (allo-HCT) is performed with curative intent for high-risk blood cancers and bone marrow failure syndromes. The efficacy of allo-HCT lies in the ability of donor T cells to mediate a potent anti-tumor response in transplant recipients, known as the graft-vs.-tumor (GVT) effect, coupled with the benefit derived from pre-transplant conditioning (1, 2). The success of allo-HCT is compromised by the development of graft-vs.-host-disease (GVHD), a complication mediated by mature donor T cells present in the graft against normal host tissue. The incidence of acute GVHD (aGVHD), a significant cause of mortality after allo-HCT, has been significantly reduced over the past decade. Transplant-related mortality has declined with the implementation of reduced intensity conditioning (RIC) regimens, new GVHD prophylaxis strategies, and the development of molecular methods aiding in early detection of viral and fungal infections in concert with modern anti-infectious agents (3–5). However, aGVHD still affects 20 to 70% of allo-HCT patients (6). Current clinical regimens for GVHD patients are primarily based on non-specific immunosuppressants for prophylaxis and treatment, such as calcineurin inhibitors or glucocorticosteroids, respectively (7). These broadly-acting agents fail to induce immune tolerance, increase susceptibility to opportunistic infections, and compromise GVT activity (8). Research in the field is focused on reducing GVHD without compromising the GVT effect. The current consensus on the initiation of GVHD pathophysiology can be divided into three primary phases:

1. ***Host tissue injury caused by conditioning regimens*** leads to the release of proinflammatory cytokines. Tissue damage from pre- transplant conditioning regimens results in a prolonged (up to 12 weeks post allo-HCT) increase of various cytokines; these include interleukin 1β (IL-1β), interleukin 6 (IL-6), interleukin 8 (IL-8), interleukin 10 (IL-10), interleukin 12 (IL-12), interleukin 21 (IL-21), interleukin 23 (IL-23), transforming growth factor β (TGFβ), and tumor necrosis factor α (TNFα) (9–11). These cytokines are primarily produced by activated dendritic cells (DCs) in response to tissue damage and microbe exposure, in concert with release of damage associated molecular patterns (DAMPs), including high mobility group protein B1 (HMGB-1) and adenosine triphosphate (ATP), as well as pathogen associated molecular patterns (PAMPs), which include lipopolysaccharide (LPS) and peptidoglycan. Both DAMPs and PAMPs can activate APCs, such as DCs and macrophages.
2. ***Donor T cell activation*** by activated APCs leads to differentiation into effector T cells, such as T helper type 1 (Th1) and T helper type 17 (Th17), both of which are pathogenic and associated with GVHD severity and mortality (12).
3. ***Effector T cell migration and target tissue destruction*** by activated donor T cells results in the initiation of GVHD (12, 13).

A myriad of cytokines, chemokines, receptors, and transcription factors are associated with T cell activation and associated inflammation, hence playing a central role in the development of GVHD. Classically, Th1 cells are believed to play a critical role in the induction of GVHD; although our group and others have demonstrated that Th17 cells also contribute (15). By targeting Th1 and Th17 specific transcription factors, T-box transcription factor TBX21 (T-bet) and Retinoic acid- related orphan receptor gamma (RORγt), respectively, it was observed that both Th1 and Th17 subsets contribute to GVHD development; yet either lineage alone is sufficient to induce GVHD (14, 15). Thus, both lineages must to be blocked in order to control GVHD. Efficacy of targeting these T cell differentiation pathways at the cytokine level are under investigation in clinical trials. Strategies for protecting/promoting prompt repair of target tissues may also reduce GVHD severity.

## Pathogenesis of Acute and Chronic GVHD

**Acute GVHD (aGVHD)** is manifested by a strong inflammatory component resulting from robust donor T cell activation and expansion. Prior to transplant, conditioning regimens involving chemotherapy and/or irradiation cause damage to host epithelial tissues, and subsequent release of danger signals such as chemokines and cytokines. The inflammatory milieu is then amplified by an activated innate immune response, consisting of APCs, natural killer cells (NK cells), neutrophils, and macrophages (13). Donor CD4 and CD8 T cell recognition of major or minor histocompatibility antigens, directly or indirectly, by host and donor APCs in conjunction with activation of the innate immune response creates a “cytokine storm” consisting of such components as interferon gamma (IFNγ), TNFα, IL-6, IL-12, and IL-23, among others (8, 16). The aforementioned combination of inflammatory factors culminates in T cell infiltration and subsequent destruction of host tissues, namely the skin, lung, liver, and gastrointestinal tract (GI tract) (16–19).

**Chronic GVHD (cGVHD)** is widely systemic and can affect essentially any of the major organ systems (8, 20). While largely undefined, the origin of cGVHD pathogenesis has been linked to thymic damage caused by conditioning, resulting in aberrant selection and subsequent release of allo/autoreactive T cells (21). Older patients receiving RIC have also been observed with cGVHD, which is potentially due to reduced thymic reserve/function (22, 23). The activation of these T cells results in cytokine production and consequential activation of macrophages and fibroblasts. Chronically stimulated donor T cells interact with bone marrow-derived B cells and produce additional factors contributing to fibroblast proliferation and activation (21, 24). In particular, T follicular helper (Tfh) cells interact with B cells via CD40L-CD40 to promote B cell proliferation, differentiation, and antibody isotype switching (25). These Tfh-B cell interactions subsequently lead to germinal center formation in which antibody diversification and affinity maturation occur, ultimately leading to an adaptive immune response (21, 24, 25). The resultant autoantibody production and tissue fibrosis lead to end organ damage (26).

## The IL-12 Family of Cytokines and Their Receptors

**Figure 1:**
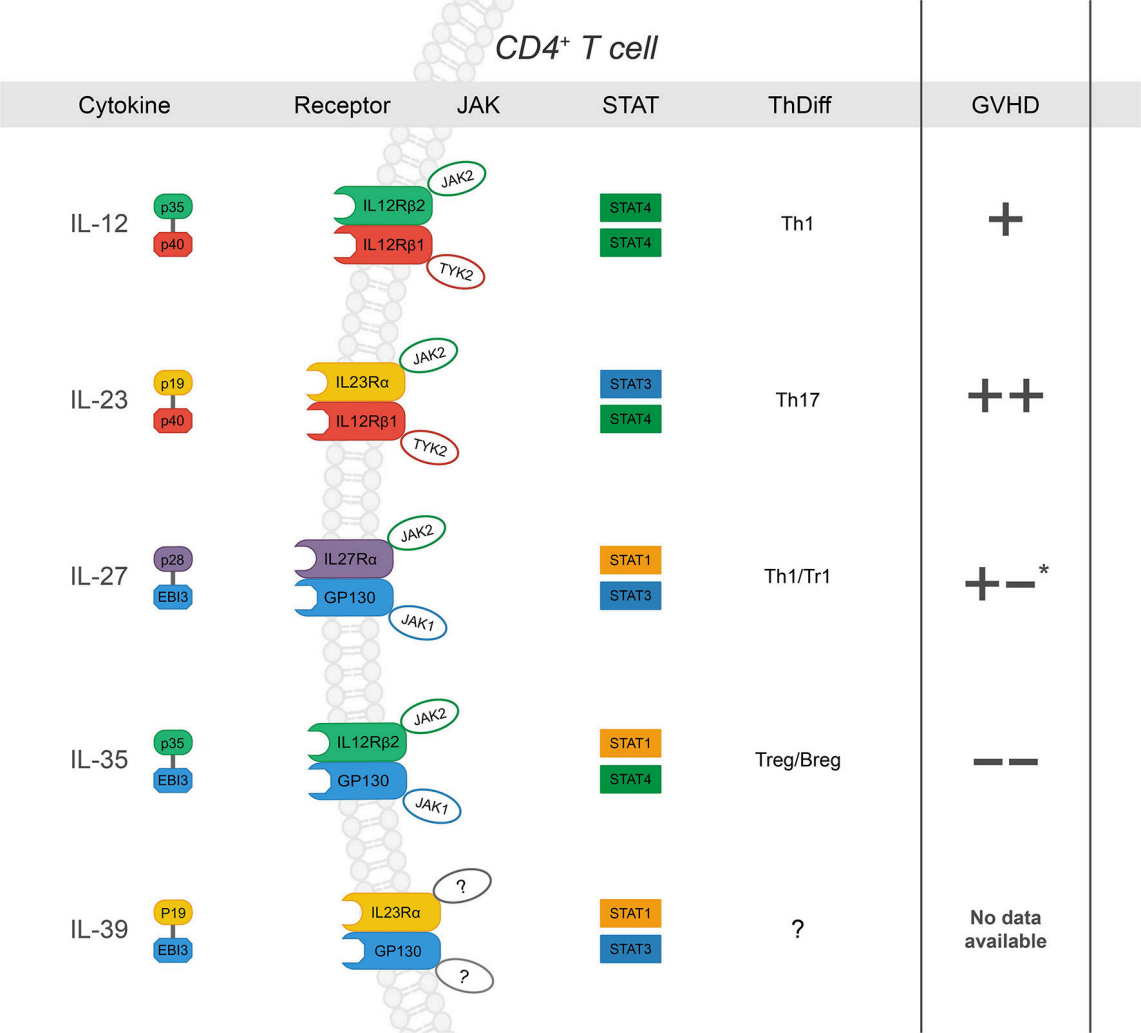
The IL-12 family: cytokines, receptors, JAK-STAT signaling, influence on CD4^+^ T cell differentiation and subsequent effect on GVHD severity after allo-HCT *(“*+” *and “–“ denote increase or decrease in GVHD severity, respectively. Listed from top to bottom)* IL-12 is a heterodimer composed of p35 and p40 (27). Upon ligation, IL-12 signals through IL-12Rβ1 and IL-12Rβ2, which form the receptor complex for IL-12 (IL-12R), subsequently leading to JAK2-STAT4 signal transduction and a positive feedback loop for Th1 differentiation (28–31). While its role in Th1 differentiation and IFNγ production has been shown to drive GVHD, there are conflicting reports concerning the specific requirement of IL-12 (32, 33); p35 can also associate with EBI3 to form IL-35, and p40 is the shared subunit with IL-23 (+). IL-23 is a heterodimer composed of p19 and p40 (34). IL-23Rα associates with JAK2 to induce primarily STAT3 phosphorylation but also STAT4 to a lesser degree (14, 35–37). IL-23 signaling results in stabilization cues for Th17 cells and has also been implicated exacerbation of intestinal GVHD (++) (38–42). IL-27, composed of p28 and EBI3, ligation to IL-27Rα/gp130 promotes IL-10 production by Tr1 cells via STAT1 at early time points post- BMT and plays a role in hindering GVHD- induced inflammation (43). However, IL-27 also inhibits Treg generation and may promote Th1 differentiation and function (+/-) (44, 45). IL-35 is composed of p35 and EBI3. IL-35 can signal through any combination of IL-12Rβ2 and gp130 was recently described as a potent immunoregulatory cytokine secreted by both T and B regulatory cells (46–48). IL-35 has been reported to suppress GVHD development. IL-39 is the most recent addition to the family and is composed of p19 and EBI3, which signal through STAT3 and STAT1. There are no reports of its function in the context of allo-HCT. *reported to promote GVHD through increased Th1 and decreased Treg differentiation; via dampen GVHD severity via Tr1 during induction phase.

The IL-12 family of cytokines can direct the donor immune response to execute a range of proinflammatory and immunosuppressive functions that are relevant in GVHD (Figure 1). They are primarily secreted by cells of myeloid origin in response to inflammatory stimuli, such as microbial products or fungal infections (49). While part of the type 1 hematopoietin family of cytokines, IL-12 family members are unique in that each member is comprised of two different subunits, or heterodimers, in which either the α or β subunit is shared among the others (46). The α-subunits include p19 (IL-23/IL-39), IL-27p28 (IL-27), and p35 (IL12/IL-35). The β- subunits include p40 (IL-12/IL-23) and Epstein-Barr virus-induced gene 3 (EBI3) (IL-27/IL-35/IL-39) (50). Further, each cytokine signals through a distinct heterodimeric receptor that is associated with its cognate subunits: IL-12R (IL-12Rβ2/IL-12Rβ1), IL-23R (IL-23Rα/IL-12Rβ1), IL-27R (IL-27Rα/gp130), IL-39R (IL-23Rα/gp130), and IL-35R (IL-12Rβ2/gp130) (46, 50). The functionality of each respective cytokine and receptor combination ranges from proinflammatory to immune suppressive in a host of pathological and physiological conditions. Yet, similar subunits and receptors involved in proinflammatory functions can also form suppressive complexes, as in the case of IL-12Rβ2, involved in IL-12 and IL-35 signaling. Therefore, deciphering the contributions of each cytokine/ receptor subunit combination is critical to understanding the immune response as a whole; the context of allo-HCT is no exception.

### IL-12/IL-12R

#### Overview of IL-12/IL-12R Signaling

IL-12 consists of p35 and p40, and acts primarily on NK cells and T cells (23, 27). IL-12Rβ1 binds to IL-12Rβ2 to form the receptor for IL-12 (IL-12R) (27, 51). Upon ligation, IL-12Rβ1 binds to Tyrosine kinase 2 (Tyk2) while IL-12Rβ2 binds to Janus Kinase 2 (JAK2). Tyk2 and JAK2 then phosphorylate tyrosine residues primarily on signal transducer and activator of transcription 4 (STAT4). Ultimately, the STAT4 complex trans locates to the nucleus and binds to the IFNγ promoter; Jun oncogene (c-Jun) is also recruited to the IFNγ promoter via STAT4 (28, 29, 52), potentiating IFNγ transcription, and Th1 differentiation. In a STAT4 -dependent manner, IL-12 also promotes expression of Interferon regulatory factor 1 (IRF1) and 4 (IRF4), transcription factors required for Th1 differentiation (53, 54). Notwithstanding the contribution of IL-12 to Th1 differentiation, IL-12/IL-12R also promotes T-cell proliferation and adhesion during activation. It has been reported that IL-12 contributes to expression of Interleukin 2 receptor α (IL-2Rα) by recruiting STAT4 and c-Jun to the promoter of IL-2R, thereby enhancing T cell proliferation (55, 56). IL-12 -induced STAT4 activation also culminates in P-selectin ligand formation, which augments T cell adhesion during differentiation (57–60). Furthermore, activation of IL-12/IL-12R signaling induces both positive and negative feedback queues which can either strengthen or reduce IL-12 signaling, respectively. For instance, STAT4 activation fosters transcription of IL-12Rβ2 and Interleukin 18 receptor 1 (IL-18R1), which cooperate to amplify IL-12 signaling and Th1 cell differentiation. While IL-12R signaling can promote proliferation via STAT5-JAK2 interactions, evidence exists that STAT5A can suppress IL-12 -induced Th1 cell differentiation through the induction of Suppressor of cytokine signaling 3 (SOCS3) (35). However, this report demonstrated that SOCS3 activity inhibits IL-12 signaling by binding to the STAT4 docking site of the IL-12Rβ2 subunit (61, 62). Hence, IL-12 is predominately associated with Th1 differentiation, yet may simultaneously hinder this effect through mobilization of STAT5A depending on the context of disease or environment.

#### IL-12 in T Cell Responses and GVHD

IL-12 promotes the differentiation of primed CD4^+^ T cells into Th1 cells, which express *Tbet*, produce IFNγ, and play a critical role in driving GVHD (15, 51). On the other hand, IL-12 negatively regulates T helper type 2 (Th2) transcription factors and associated cytokine production (63). As such, IL-12Rβ2 expression is absent on Th2 cells but upregulated in Th1; an increase in Th2 differentiation is associated with reduced acute GVHD yet can exacerbate chronic GVHD (64, 65). In addition, CD40-CD40L interactions between T cells and APCs can fuel IL-12 production by APCs, which amplify innate immune cell responses through IFNγ production (66). With regards to the IL-12 cytokine itself, the pool of available data is somewhat contradictory in the context of aGVHD. IL-12 has been reported to drive GVHD due to its stimulatory effect on Th1 cells (67, 68). IL-12 serum levels in aGVHD patients are increased compared to healthy controls, yet no correlation between higher grade GVHD (II-IV) and IL-12 has been observed (69) (Table 1). Conversely, exogenous IL-12 administration was suggested to be protective in GVHD via an IFNγ-dependent mechanism (72). Previous studies observed that a single injection of IL-12 at the time of allo-HCT stifles GVHD in myeloablative-conditioned recipients (32, 33, 72). The protective or pathogenic role of IL-12 seemingly relies on the dose and timing IL-12 injection, and irradiation type for the recipient conditioning regimen in murine BMT models (73). In NK cells, IL-12 induces cytotoxic events through STAT4 and subsequent activation of the Perforin 1 (perforin) gene promoter (74). A recent report a describes IL-12/IL-18 activated donor NK cells mitigate GVHD but enhance GVT activity (75).

**Table 1:** Expression levels of IL-12 family cytokines in aGVHD patients.

**aGVHD grade**	**0–1**	**2–4**	**References**
IL-12	↑	↑	(69)
IL-23	↑	↑↑	(40)
IL-27	↓	↓	(70)
IL-35	↓	↓↓	(71)

*Representative table of IL-12, IL-23, IL-27, and IL-35 levels detected in the serum of patients with aGVHD. Upward arrows indicate increases compared to healthy donors, while downward arrows indicate decreases*.

Apart from advocating Th1 responses, IL-12 plays a critical role in T follicular helper cell (Tfh) differentiation and function through STAT4 and Tbet (76, 77). Consistent with the crucial role of Tfh cells in cGVHD pathogenesis, administration of anti-p40 mAb in recipient mice significantly reduced Tfh generation and scleroderma manifestations of cGVHD after allo-HCT^57^. Thus, targeting one or more of the IL-12 cytokine/receptor subunits represents a promising therapeutic strategy to reduce cGVHD.

### IL-23/IL-23R

#### Overview of IL-23/IL-23R Signaling

IL-23 consists of p19 and p40. The IL-23R is a heterodimer comprised of IL-12Rβ1 and IL-23Rα. IL-23R associates with JAK2 and, in a ligand-dependent manner, with STAT3. IL-23- induced activation of STAT3 leads to direct binding of phosphorylated STAT3 to IL-17A and IL-17F promoters. STAT3 up-regulates the expression of RORγt, the master transcription factor of Th17, which is critical for the expression of two members of the Interleukin-17 family, IL-17A and IL-17F (78–80). SOCS3 inhibits JAK2 activity, hence decreasing IL-17A and IL-17F expression (78).

IL-23 signaling regulates Th17 cells. IL-23 plays an important role in expanding and maintaining the Th17 cell population, a T cell subset involved in homeostatic antimicrobial immune responses as well as in the propagation of many autoimmune diseases (81). IL-23 is an indispensable factor for promoting pathogenicity of Th17 cells, yet is not required for initial differentiation (82–85). IL-23 has been shown to control Th17 responses through regulating T cell metabolism. TCR stimulation induces GLUT-1 surface expression and subsequent lactate production, promoting glucose uptake (86, 87). T cells under Th17 polarizing conditions undergo a HIF1-α- dependent metabolic switch to glycolysis, and data indicates that IL-23 might contribute to this effect via PKM2 and HIF1-α (88). Notably, allogeneic T cells were shown to depend on glycolysis for effector function during GVHD development, yet a connection to IL-23 and glycolysis has not been demonstrated. HIF1-α induction has also been associated with IL-23 production in dendritic cells; this link between HIF1-α and PKM2 has been previously established in cancer cells (89–91). Therefore, PKM2 may possess more than one function in addition to its role in glycolysis manifested by transcriptional activation as a protein kinase (92). Taken together, IL-23 signaling through PKM2/STAT3 may directly contribute to the metabolism of Th17 cells and, in concert with IL-6, could represent an essential factor for lineage commitment (93). Glucocorticoid-induced protein kinase 1 (SGK1) is critical for IL-23R expression through deactivating murine Foxo1, which directly represses IL-23R expression (94). SGK1 is essential for the induction of pathogenic Th17 cells and implicates environmental factors, such as a high-salt diet, as triggers to Th17 development and subsequent tissue inflammation (95). Lastly, while Blimp-1 IL-23-dependent Blimp-1 enhances Th17 pathogenic factors such as GM-CSF and IFNγ, and co-localizes with RORγt and STAT-3 at *Il23r, Il17a*, and *Csf2* enhancer sites (96).

#### IL-23 Signaling in Autoimmune Diseases

Studies show IL-23 signaling contributes to the pathogenesis of various autoimmune diseases. In mice, it was demonstrated that bacteria-driven innate colitis is associated with an increased production of IL-17A and IFNγ in the colon. Stimulation of intestinal leukocytes with IL-23 induced the production of IL-17 and IFNγ exclusively by innate lymphoid cells expressing IL-23R, which were demonstrated to accumulate in the inflamed colon. These results identified a previously unrecognized IL-23-responsive innate lymphoid population that mediates intestinal immune pathology and may therefore represent a target in inflammatory bowel disease (97–99). Intestinal IL-23-responsive innate cells are also a feature of T cell-dependent models of colitis, which resembles many of the features seen in intestinal GVHD with respect to T cell infiltration resulting in inflammation and gut injury. The transcription factor RORγt controls IL-23R expression, as it was shown that Rag/Rorc-null mice failed to develop innate colitis which is dependent on IL-23 (98). In addition, expression of IL-23 and IL-23R was increased in the tissues of patients with psoriasis (100). Injection of a neutralizing monoclonal antibody to IL-23p19 in a xenotransplant mouse model showed IL-23-dependent inhibition of psoriasis (100).

#### IL-23 Signaling in GVHD

Our group has demonstrated both Th1 and Th17 subsets are required to induce GVHD (15). Pharmacological inhibition of IL-23p19 results in reduced GVHD, and recent evidence suggests that IL-23R drives GVHD pathogenesis (38–41). These studies show that a CD4^+^CD11c^+^IL-23R^+^ T cell population induces colonic inflammation during GVHD, indicating a key role for IL-23R expression on donor T cells in mediating damage to the gut after allo-HCT. Consistently, the gene expression levels of *IL-23* and *IL-23R* were upregulated in murine colons after allo-HCT (38). These studies demonstrate that the colon is specifically protected via IL-23p19 signaling blockade, and that GVL activity is maintained. In a patient cohort, Liu et al. observed IL-23 mRNA expressions in patients with aGVHD were significantly higher than those in healthy donors, and IL-23 and IL-23R expression were positively correlated with IL-17 expression (101). These studies additionally showed that IL-23 serum levels were elevated during the onset of aGVHD, yet decreased during disease remission (Table 1). In aGVHD, two out of three independent studies in patients found that a single nucleotide polymorphism (rs11209026) in IL-23R of donor origin reduced incidence of GVHD; the third study did not observe any effect (19, 102). Hence, blocking either p19 or p40 reduces aGVHD and IL-23R deficiency in donor T cells results in abrogated GVHD. These results indicate IL-23 also plays a key role in GVHD pathogenesis. Albeit, a recent paper demonstrated genetic inactivation of IL-23R, or the transcription factor RORγt, within donor T cells similarly ablated Th17 cell formation *in vivo* but preserved the T cells' ability to induce intestinal GVHD in an indistinguishable manner compared to wild-type controls (103).

#### Developing New Strategies to Target IL-23R

The crystal structure of IL-23Rα was recently reported (104). Hence, development of pharmaceutical compounds capable of specifically binding/inhibiting the IL-23R has been stagnant since its discovery in 2002. It appears that IL-23 binds IL-23R with an affinity of 44 nM, while binding IL-12Rβ1 with an affinity of 2 μM; nonetheless, the affinity of the IL-23:IL-23R complex for IL-12Rβ1 has been described as 25 nM, despite no apparent interaction of IL-23R with IL-12Rβ1, implying that there is a cooperative effect which is likely to be due to a conformational change of IL-23 upon binding IL-23R, which is indeed observed crystallographically (104–108). In a recent publication, hydrogen–deuterium exchange mass spectrometry (HDX-MS) was used to demonstrate IL-23 binding to the N-terminal immunoglobulin domain of IL-23R in both the solid state as well as under more physiologically relevant conditions. This data allowed specific identification of a binding epitope using a macrocyclic small molecule against IL-23R for the first time (106). However, IL-23R antagonism is not a new concept, as a peptide antagonist was shown to reduce inflammation in different models of autoimmune disease (109). The aforementioned data presents exciting new possibilities for future studies, yet efficacy of such prototype molecules requires vigorous testing in preclinical models.

### Interplay Between IL-12 and IL-23

Recent findings have emphasized the need to develop therapeutics methods that enable targeting of IL-12 and IL-23 signaling. Interestingly, not only do the cytokines IL-12 and IL-23 share the same cytokine subunit, p40, but also the cognate receptor, IL-12Rβ1. Thus, these shared motifs provided the rationale for blocking Th1 and Th17 responses simultaneously through targeting p40/IL-12Rβ1. However, p40 itself has a diverse set of functions. For example, p40 has a chemo attractant role for macrophages mediated by IL-12Rβ1 alone, which is dependent on the intracellular domain of IL-12Rβ1 to signal; these reports were published with regard to IL-12Rβ1 signal transduction in response to a p40 homodimer (110, 111). With respect to alloreactive T cells, this p40 homodimer was demonstrated to have antagonistic activity for CD4^+^ IFNγ production, yet could amplify IFNγ production by CD8^+^ T cells (112). Hence, targeting p40 in the context of GVHD may result in enhanced Th1 responses and potentially hinder CD8 mediated GVT responses (113). IL-12Rβ1 promiscuity among the IL-12 family has both assisted in the development of pharmaceuticals to target both pathways (as in the case of p40), yet also illuminated their complexity. Th1 and Th17 differentiation and stability converge at IL-12 and IL-23 signaling, respectively, as both signaling motifs share p40 at the cytokine level and IL-12Rβ1 for downstream signal transmission. Supported by studies done in mice and men, IL-12 is documented to induce IFNγ production by Th17 cells with respect to cytokines in the milieu, *in vivo* and *in vitro*, respectively (114, 115). This Th1/Th17 subset was shown in Crohns disease (114).

#### p40 and GVHD

Targeting p40, a shared subunit of IL-12 and IL-23 cytokines, consistently mitigates GVHD in clinical and preclinical studies. Our group and others have demonstrated that neutralization of the p40, using genetically deficient mice and pharmacological inhibition, alleviated acute and chronic GVHD in murine models through reducing Th1 and Th17 differentiation (116, 117). Recent data from Pidala et al. demonstrates *in vivo* IL-12/IL-23p40 neutralization with ustekinumab blocks the Th1/Th17 response and improves overall survival in patients after allo-HCT (118). Notably, in other models of autoimmunity, in which Th17 is the major mediator of diseases, much of the originally allocated inflammatory actions of IL-12 have since been shown to be influenced by IL-23, as many studies prior to IL-23 identification were conducted via targeting p40 (84, 119). Therefore, future studies should focus on the biological differences of IL-12 and IL-23 in order to determine why IL-12 can exacerbate GVHD in some contexts yet suppress it in others, yet pharmacologically targeting p40 can be efficacious in reducing GVHD severity in experimental and clinical settings.

#### IL-12Rβ1 Deficiency in Human Diseases

IL-12Rβ1 was identified in 1994 by Chua et al. as a member of the hemopoietin receptor superfamily, an amino acid type I transmembrane protein that resembled the IL-6 signal transducer, gp130 (120). It was not until 1996 that IL-12Rβ2 was identified, which subsequently led to the identification of a high affinity IL-12 receptor complex when IL-12Rβ1 and IL-12Rβ2 were coexpressed (27). Notably, the existing data with respect to IL-12Rβ1 in murine models of GVHD is sparse, although there is an abundance regarding its role in conferring immunity to mycobacteria and other infections (121–123) in human. However, given that deficiency of IL-12Rβ1 is relevant in patients, there are a plethora of case studies documenting related T cell responses (124). The IL12Rβ1 promoter, when deficient of the −265 to −104 region, suggested the existence of an important regulatory element. Furthermore, the −111A/T substitution appeared to cause decreased gene transcriptional activity, such that cells from−111A/A individuals were observed to have increased IL12Rβ1 mRNA levels compared with those from−111T allele carriers. Thus, in individuals with the−111T/T genotype, reduced IL12Rβ1 expression may lead to augmented Th2 cytokine production in the skin, and subsequently contribute to the development of atopic dermatitis and other associated allergic diseases (125).

Of particular interest is the role of IL-12/IL-12Rβ1 pathway in the induction of highly suppressive antigen-specific Th1-like Tregs from naïve Tregs (126). It was recently described that, in two patients with IL-12Rβ1 deficiency, features of systemic autoimmunity, and photosensitivity were observed (127). These features are similar to transgenic mice deficient for IL-12Rβ2, which develop an autoimmune syndrome consisting of anti-DNA positivity, immunocomplex glomerulonephritis, and multiorgan lymphoid infiltrates with features of vasculitis. However, IL-12Rβ1 deficient patients displayed substantially less circulating memory Tfh and memory B cells than healthy controls (77). In humans, TGFβ cooperates with IL-12 and IL-23 for expression of Tfh molecules: CXCR5, ICOS, IL-21, and the transcriptional regulator Bcl6 (128). Hence, data taken from studies in IL-12Rβ1 deficient patients suggests a regulatory role for IL-12, perhaps derived from Treg function, which may explain the contradictory results observed in murine models. Albeit, the role of IL-12 in Tfh/B cell axis seems at baseline consistent among experimental and clinical studies. While the aforementioned discrepancies are preliminary in comparison to the mass of studies documenting proinflammatory roles of IL-12, there is still much to learn in terms of IL-12 function; especially with respect to differences vs. IL-23.

### IL-27/IL-27R

IL-27 is comprised of IL-27p28 and EBI3, binds to IL-27Rα/gp130, and is the only member of the family that is not secreted as a functional dimer (129). In fact, IL-27p28 is also known as IL-30. As such, the biological mechanisms associated with the role of IL-27 vs. IL-30 in the immune response are ambiguous, displaying both proinflammatory and suppressive functions that seem to be dependent on the disease model. IL-30 was previously reported to antagonize IL-27-mediated proinflammatory responses (130). Further, IL-30 inhibited activity by IL-6, IL-11, and IL-27 in the absence of EBI3 through gp130 binding (131). These findings support a role for IL-30 in hindering proinflammatory effects by such cytokines as IL-6. Yet, a recent report demonstrates that pharmacological blockade of IL-27p28 alleviates GVHD in mice, and resulted in augmented Treg responses (45). Consistently, our group found that IL-27Rα expression promotes T cell pathogenicity during GVHD induction, and was attributable to augmented Th1 effector function (44). However, a report by Zhang et al. elegantly demonstrated the function and prevalence of Tr1 early after allo-BMT; noting a significant role in IL-10 production which could ameliorate GVHD and which was dependent on IL-27 (43). Tr1 cells differentiate in the presence of IL-27 and are the central cell type implicated in IL-27- related suppressive activity, producing IFNγ, and IL-10 simultaneously. Of note, experiments performed in the aforementioned report depleted Tregs in donor grafts before transplantation, and therefore may be the reason they saw no difference compared to similar models used in studies by Belle et al and those by our group. These studies can be connected in such a way that IL-27 promotes Tr1 cells early after BMT and can decrease GVHD independent of Tregs; yet in later stages IL-27 inhibits iTregs and Th2 cells and promotes Th1 differentiation; this ambiguous pattern is very similar to that seen in models of autoimmunity. Clinically, a study by Odile et al. demonstrated that membrane IL-27Rα existed in a soluble form (sIL-27Rα), functioning as a natural antagonist of IL-27, in healthy human serum, as well as in the serum of patients with Crohn's disease, suggesting that sIL-27Rα may play an immunoregulatory role in normal as well as pathological conditions (132). In extended studies by Liu et al. sIL-27Rα was identified as a potential biomarker for the development of aGVHD (70). However, IL-27 levels have only been shown to positively correlate with sIL-27R, which Liu et al. suggested was protective in GVHD. Higher levels of serum sIL-27Rα correlated with lower grade aGVHD, however, did not show any correlation for the prediction of cGVHD (70). In future investigations, studies should focus on how IL-27 blockade modulates established GVHD, which is characterized by Th1–mediated inflammation in the skin, gut, and liver. Additionally, it remains to be determined whether Treg cells deprived of Tbet induction by IL-27 will possess the transcriptional machinery sufficient to infiltrate into active GVHD sites. It will also be critical to determine the effects of IL-27 blockade on the GVT effect.

### IL-35/IL-35R

IL-35 consists of p35 and EBI3, and functions as a regulatory cytokine released by CD4^+^Foxp3^+^ T regulatory cells (Tregs), as well as regulatory B cells (Bregs), to suppress inflammation, and subsequently reduce the severity of autoimmune diseases (50, 133). Interestingly, IL-35 signaling in Tregs was transduced via receptor combinations of IL-12Rβ2/gp130, IL-12Rβ2/IL-12Rβ2, or gp130/gp130, none of which could be clearly identified as the high affinity receptor (71). Currently, the suppressive effect of IL-35 in mouse models of aGVHD have been established by Zhang et al. (134) and Liu et al. (47). Importantly, IL-35 levels in the serum of aGVHD patients was significantly decreased in higher grade GVHD (II-IV) compared to lower grade (0-I) (47) (Table 1). Liu et al. (47) Collectively, these studies demonstrate that IL-35 is associated with higher frequencies of Tregs, reduced Th1 differentiation, reduced GVHD when combined with rapamycin, and evidence indicating maintenance of GVL activity (47, 134). However, IL-35 within the tumor microenvironment may oppose T cell responses required for GVT response by inducing effector exhaustion (135). Given the potential regulatory effects mediated by IL-35, we speculate the cytokine may be relevant in controlling cGVHD. Further, a recent publication by Yin et al. demonstrated that IL-35 administration skewed T cell differentiation from Th17 to Treg in islet cell transplantation models (136). IL-35 is clearly immunoregulatory and potentially useful in GVHD prevention, but a better understanding of its impact on T cell anti-tumor responses is needed prior to clinical translation.

### IL-39/IL-39R

IL-39 has been proposed to consist of p19 and EBI3, and is the most recent addition to the family (137). Wang et al. published the first report describing the function of an additional heterodimer that involves p19 complexed with a subunit other than p40. IL-39 is secreted by activated B cells and was demonstrated to be significantly elevated in lupus models compared to other IL-12 members using MRL/lpr mice (137). The receptor for IL-39 was determined to be formed by dimerization of IL-23R and gp130 and signal through STAT1 and 3. While associated with neutrophil differentiation and expansion, the proinflammatory effects of IL-39 have yet to be fully defined. In a different report by Ramnath et al. IL-39 was shown to be secreted by keratinocytes and contribute to wound healing (138). While the function of IL-39 may be context dependent, these disparate reports indicate that IL-39 may also act on a broad range of cell types. Hence, further clarification regarding the general role of IL-39 in immunity is required in order to determine its effect in GVHD.

## Interplay Between IL-6 and IL-12 Super Families

While promiscuity among IL-12 cytokine and/or receptor family is a common theme, the degree of association with glycoprotein 130 (gp130), better known for its role in IL-6 signaling, has become an intriguing area of research. Gp130 forms the link between “IL-6R/IL-12R” families, which collectively include Leukemia Inhibitory Factor Receptor (LIF-R), IL-12Rβ1, IL-12Rβ2, Granulocyte Colony-Stimulating Factor Receptor (GCSF-R), and Oncostatin-M Receptor (OSM-R); and serves as a shared signal-transducing subunit for IL-6, IL-11, Leukemia Inhibitory Factor (LIF), Oncostatin-M (OSM), Cilliary Neurotrophic Factor (CNTF), Cardiotrophin-1 (CT-1), Cardiotrophin-like cytokine (CLC), and IL-27(139, 140). Importantly, gp130 is well-documented for its capacity to transduce signals, especially for IL-6, a staple cytokine involved in inflammation. The complex of IL-6 and IL-6R binds to the ubiquitously expressed receptor subunit gp130, which forms a homodimer and thereby initiates intracellular signaling via the JAK/STAT and the MAPK pathways. IL-6R expressing cells can cleave the receptor protein to generate a soluble IL-6R (sIL-6R), which can still bind IL-6 and can associate with gp130 and induce signaling even on cells, which do not express IL-6R. This paradigm has been called IL-6 trans-signaling whereas signaling via the membrane bound IL-6R is referred to as classic signaling (139–141).

## Translational Potential for Targeting the IL-12/IL-12R Family

### Targeting IL-12 and IL-23 Cytokines

Regarding clinically translatable approaches targeting the IL-12 family, ustekinumab targets the p40 shared subunit between IL-23 and IL-12. Ustekinumab added to tacrolimus and rapamycin was shown to be safe and effective for GVHD prophylaxis after related or unrelated allo-HCT. In a randomized, blinded, placebo-controlled study, ustekinumab significantly improved overall survival, and CRFS (Conditional Random Fields Score), a novel composite endpoint including moderate/severe cGVHD and relapse-free survival (118). Guselkumab and tildrakizumab, two monoclonal antibodies against p19, approved for treatment of plaque psoriasis (142, 143). However, these specific neutralizing antibodies against p19 have yet to be evaluated in GVHD patients. While inhibiting JAK2 signal transduction by IL-12 and IL-23 is a promising strategy, the question pertaining to how the shared or disparate receptors contribute to signal transduction and the consequential effect on T cell differentiation in allo-HCT remains unclear. The advancement of targeted pharmacological compounds specific for IL-12 or IL-23 signaling will be required to adequately dissect these scientific questions appropriately across species.

### Targeting IL-12 Family and Infection

The use of any immunosuppressive agent carries the theoretical risk of impairing host defense responses to pathogens and/or decreased tumor surveillance. Relative risks of targeting IL-12 and/or IL-23 are well-documented with respect to potential risk of infections. When challenged with *Mycobacterium, Salmonella* or *Candida*, mice lacking IL-12p35, IL-12p19, and IL-12/23p40 have phenotypes that generally mirror what has been observed in humans. As mentioned earlier, studies of IL-12/23p40 and IL-12Rβ1 deficiencies indicate that human IL-12 and IL-23 are redundant in host defense to many pathogens. Importantly, allo-HCT recipients treated with ustekinumab did not experience any increase in opportunistic infections or reactivation of CMV, EBV, or HHV6 compared to the placebo arm (118).

## Downstream Signaling by the IL-12/IL-12R Families and Relevant Translational Potential

### Jak2: The Center of IL-12/IL-12R Family Signaling

Both IL-12R and IL-23R have been demonstrated to signal via JAK2; JAK2 deficient donor T cells or JAK2 inhibition with pacritinib were demonstrated to significantly alleviate GVHD in murine models via spared Treg differentiation and reductions in Th1 and Th17 differentiation in in mouse and human T cells (144). This is consistent with reports describing a common reliance on JAK2 by both IL-12 and IL-23. A key difference in downstream signaling is that IL-12 phosphorylates primarily STAT4, while IL-23 mainly induces STAT3 phosphorylation. Betts et al. reported that at 20 days' post allo-HCT, pSTAT3 was significantly increased in CD4^+^ T cells among patients who would later develop aGVHD; a signaling pathway known to directly drive the transcription of Th17 lineage-specific genes (14).

JAK2 signal transduction is implicated in human autoimmune syndromes and GVHD. IL-6, IL-12, and IL-23 mediate inflammation and activate T cells via JAK2 (14, 145–148). Blocking the IL-6 receptor with the monoclonal antibody tocilizumab has demonstrated efficacy in a phase II GVHD prevention trial (149). Tocilizumab, however, does not fully impair pathogenic Th1/Th17 responses (150), which may be attributed to the IL-12 and IL-23 receptor signaling-induced JAK2 activation to promote Th1 and Th17 differentiation, respectively. Neutralization of these p40 cytokines prevents GVHD in murine models, and may have activity in treating patients with steroid refractory GVHD (151).

### JAK2 Inhibition in GVHD

JAK2 inhibition is an alternative approach to suppress IL-6 and p40 receptor signal transduction and induce durable tolerance to alloantigens. JAK2 inhibitors are clinically efficacious in myelofibrosis, a hematological disease often driven by constitutive JAK2 activation (152). The existing evidence regarding JAK2 as a therapeutic target for acute GVHD is primarily supported by observations using ruxolitinib, an equimolar inhibitor of JAK1 and JAK2 (153–156). Ruxolitinib has been previously demonstrated as efficacious in treating steroid-refractory GVHD, and is clearly immunosuppressive. In part, JAK1 mediates the biologic effects of common gamma chain cytokines, including IL-2 and IL-15. Ruxolitinib suppresses host-reactive T cells in mice and humans. Although not observed in murine transplant studies, ruxolitinib treatment reduces the quantity of Tregs as well as the beneficial effects of NK cells in myelofibrosis patients (157–159). Therefore, a JAK2 inhibitor has the potential to prevent GVHD without conceding JAK1-mediated functions provided by donor lymphocytes. Further research determining the differential effects of JAK1 and JAK2 is required to resolve these conundrums.

Given the recent discovery of IL-39 (p19/EBI3) and its cognate receptor (IL-23Ra/gp130), the question pertaining to the individual requirement for each particular cytokine/receptor complex becomes much more complex. IL-39R was shown to signal via STAT1/ STAT3 pathways, which overlaps with IL-27 and IL-23 signaling, respectively. The manner by which IL-39R and IL-23R on T cells may differentially or similarly impact the T cell response in allo-HCT requires further investigation.

## Conclusion

The interplay between IL-6/IL-12 family members pertaining to T cell differentiation requires further investigation in the field of allo-HCT. Specific neutralizing antibodies against receptor subunits, such as IL-23Rα, are in development but have yet to be evaluated in preclinical models. While inhibiting JAK2 signal transduction by IL-12 and IL-23 is a promising strategy, the question pertaining to how the shared or disparate receptors contribute to signal transduction and the consequential effect on T cell differentiation in allo-HCT remains unclear. The advancement of targeted pharmacological compounds specific for IL-12 or IL-23 signaling will be required to adequately dissect these scientific questions appropriately across species.

## Author Contributions

DB wrote the manuscript. YW, X-ZY, and BB edited and revised the manuscript.

### Conflict of Interest Statement

The authors declare that the research was conducted in the absence of any commercial or financial relationships that could be construed as a potential conflict of interest.
